# Impact of recycled manure solids bedding on hygiene and odds of hock lesions in dairy cows

**DOI:** 10.3389/fvets.2022.1061632

**Published:** 2022-12-23

**Authors:** Annie Fréchette, Gilles Fecteau, Simon Dufour

**Affiliations:** ^1^Regroupement FRQ-NT Op+Lait, Saint-Hyacinthe, QC, Canada; ^2^Mastitis Network, Saint-Hyacinthe, QC, Canada; ^3^Department of Pathology and Microbiology, Faculty of Veterinary Medicine, Université de Montréal, Saint-Hyacinthe, QC, Canada; ^4^Department of Clinical Sciences, Faculty of Veterinary Medicine, Université de Montréal, Saint-Hyacinthe, QC, Canada

**Keywords:** dairy, bedding, recycled manure solids, hock lesions, hygiene

## Abstract

The use of recycled manure solids (RMS) as bedding for dairy cows has become more popular over the last decade. Once producers own the RMS producing equipment, they are not restricted in the amount of bedding they can use in stalls, due to its large availability and low production costs. Thus, they are usually able to provide a soft lying area for their cows. Nevertheless, the potential positive impact of RMS on cows' hygiene and odds of hock lesions is not clearly demonstrated. Objectives of this research were, therefore, to compare RMS-bedded cows' hygiene level and odds of hock lesions to straw-bedded cows (comparative group). To achieve this, an observational cross-sectional study was conducted in 27 RMS- and 61 straw-bedded herds. During the farm visit, 30 cows per herd were scored for hygiene of three body regions (udder, lower legs and flank/upper legs) using a score ranging from 1 to 4 (1 being the cleanest and 4 the dirtiest). In addition, both hocks were scored (0 to 3) to evaluate the presence of lesions (0 when there was no lesion and 3 when a swelling protrusion > 2.5 cm was present). Continuation-ratio models were used to compute the odds of having a given score to the odds of having a lower score. Recycled manure solids usage was associated with lower odds of having an udder hygiene score ≥3 [odds ratio (OR): 0.43; 95% CI: 0.20, 0.95] and of having a score of 4 (OR: 0.29; 95% CI: 0.09, 0.93). It was also associated, compared to straw, with cleaner lower legs. We observed lower odds of having a score ≥2 (OR: 0.45; 95% CI: 0.21, 0.98), ≥3 (OR: 0.16; 95% CI: 0.04, 0.66), or a score of 4 (OR: 0.07; 95% CI: 0.01, 0.40) in RMS-bedded herds. No statistically significant association could be highlighted between RMS usage and flank/upper legs hygiene. Furthermore, we did not observe any statistically significant associations between bedding type (RMS vs. straw) and odds of hock lesions. In conclusion, cows bedded with RMS had generally cleaner udders and cleaner lower legs than straw-bedded ones. No association was found between bedding type and odds of hock lesions.

## Introduction

The choice of using a certain type of bedding in dairy herds is driven by several factors. Bedding must be compatible with the farm's equipment, available in large quantities and at a reasonable cost. From the animal's perspective, an appropriate bedding should allow the expression of normal behavior, without any risk of slipping (1). Bedding should also contribute to hygiene by its absorption capacity and should provide a soft resting area (2, 3). Recycled manure solids (RMS) bedding seems promising due to its ease of installation, although production equipment is costly, in all types of farms (tie-stall vs. free stall) and the large quantity available, once the production cycle is initiated (4, 5). In Quebec, RMS bedding is mostly produced by maturation in heap or in closed container of the solid manure fraction (6). Producers may also let the solid fraction mature in a rotating drum or use it “green” (i.e., use the solid fraction immediately after the separation process). Finally, they may process the manure trough a biodigester followed by a separation process. Drying devices are not currently used in Quebec's herds as part of the production process (6).

Studies on association between use of RMS bedding and animal health are scarce and concern mainly udder health. Recycled manure solids bedding has not been associated with an increased incidence of subclinical mastitis (4, 7, 8). The effect of RMS bedding on incidence of clinical mastitis is less clear, with a study reporting RMS usage as a significant risk factor for clinical mastitis (7) while others did not (6, 9, 10). In a recent study (6), however, it was reported that cows housed on RMS bedding had a 7 times greater risk of experiencing a clinical mastitis due to *Klebsiella pneumoniae*. On the other hand, the effect of RMS bedding on cows' hygiene and odds of hock lesions remains to be measured.

Lombard et al. (11) evaluated cows hygiene in a large cross-sectional study (297 free stall American Holstein herds; 28 herds using RMS as bedding) and concluded that severe soiling, defined as cows having large amounts of manure on legs, udder or flanks, was not associated with bedding type. In another study, they observed that hygiene scores for legs, udder and flank were similar between primiparous cows bedded with sand and cows on deep-bedding RMS system (12). There was a significant difference, however, when these two beddings were used on mattresses (shallow bedding). In such a system, cows on RMS bedding had a poorer hygiene level. Finally, Patel et al. (10) did not detect a difference in udder hygiene score of RMS-bedded cows compared to animals bedded with other organic materials (shavings, straw and other products).

During their study, Lombard et al. (11) concluded that bedding type was associated with severity of hock lesions (hair loss, swelling or draining lesions). Sand bedding (their reference bedding) was associated with the lowest percentage of severe hock lesions when compared to RMS bedding (0.7 vs. 2.7%). Cows housed on RMS, in contrast with sand-bedded ones, had a severe hock lesion incidence rate ratio of 3.75 while straw-bedded cows had an incidence rate ratio of 2.60. Similarly, it was demonstrated in another study that use of sand bedding was associated with a lower prevalence of hock lesions (mild/moderate/severe) than RMS bedding (13). However, these latter authors found no statistically significant difference in the prevalence of severe hock lesions when comparing RMS bedding to sand. In that latter study, incidence of hock lesions were 3.44 higher in RMS herds vs. sand-bedded herds and 9.25 higher for straw herds vs. sand-bedded herds. A difference in hock lesions prevalence between cows bedded with RMS and sand-bedded ones was also observed by Esser et al. (12). However, the greater hock scores observed in RMS-bedded cows in that latter study were, again, entirely associated with use of mattresses (in contrast with deep bedding stall systems). Prevalence of hock lesions and severe hock lesions of cows housed on deep-bedding RMS was also estimated by Husfeldt and Endres (14) at 49.4% (95% CI: 45.4, 53.4) and 6.4% (95% CI: 5.6, 7.3), respectively, in contrast with 67.3% (95% CI: 62.4, 71.9) and 13.2% (95% CI: 11.8, 14.7) in shallow RMS bedding.

In summary, in the available literature, effect of RMS bedding on cows' hygiene is reported to be comparable with other bedding materials. However, association of this product with hock lesions is not clear. Recycled manure solids bedding seems to be associated with higher risk of hock lesions than sand, but when compared to straw, may be more or less beneficial for cows. Finally, it is important to note that bedding thickness may have influenced the results of the aforementioned studies.

Considering this lack of knowledge, our objectives were, therefore, to describe the association between use of RMS bedding and (1) cows' hygiene, and (2) odds of hock lesions, when compared to straw bedding. Straw bedding was the most commonly used bedding product in Quebec at the time. In general, particle size of straw bedding is highly variable from one farm to another, and straw is usually added on top of mattresses or rubber mats. We hypothesized that both animal groups would have the same hygiene level and, for the same farm-bedding strategy (tie-stall vs. free stall, shallow vs. deep bedding), would present the same odds of hock lesions. This paper is part of a larger project where we studied many aspects of RMS bedding: parasitic load and survival (15), bacteriological content and microbiota (16), bulk milk quality (17), pathogen-specific clinical mastitis incidence (6) and subclinical mastitis incidence (8).

## Materials and methods

This project was approved by the Animal Care and Use Committee of the Faculté de Médecine Vétérinaire (Université de Montréal; protocol 17-Rech-1886). An observational cross-sectional study design was chosen to evaluate dairy cows' hygiene level and odds of hock lesions.

### Herd recruitment

We aimed at recruiting 30 cows/herd from 90 herds, of which ≥20 would be using RMS bedding for lactating cows, and the remainder would be using straw bedding (as a comparative group). This number and ratio were determined by *a priori* power estimations which were conducted for the various outcomes studied (bedding microbiota, (16); parasitic load, (15); clinical mastitis incidence, (6); bulk tank milk quality, (17); subclinical mastitis (8), hygiene, and hock lesions). The number of farms to recruit was determined mainly by the clinical mastitis outcome, which required the largest sample size (see Frechette et al. (6) for details). In the case of hygiene or hock lesions, we computed a power >0.80, when using 30 cows/herd from 20 RMS and 70 straw-bedded herds to detect differences corresponding to OR>1.5 (or OR <0.67) when the reference proportion of diseased animals was 0.10 (note that a greater study power was achieved with larger reference proportion of diseased animals or with more than 20/90 RMS herds).

A list of potential RMS herds was assembled with the help of equipment dealers, veterinarians, dairy producers and through social media. Straw-bedded herds were offered to participate in the project through Dairy herd improvement association (DHIA; Lactanet, Ste-Anne de Bellevue, QC, Canada). To be eligible, producers needed to be within 250 km of the research facilities (Saint-Hyacinthe, QC, Canada) and to have used the same bedding for at least 6 months prior to the time of the visit.

Potential participants were contacted between July and December 2017 to verify their eligibility and willingness to participate to the study. If they were not eligible, basic demographic data such as herd size and RMS processing methods were collected to evaluate potential selection bias.

### Data collection

At the time of the visit, a questionnaire related to bedding management was completed with the producer, the buildings were visited, and various samples (milk, feces, bedding) were collected for other parts of the study (6, 8, 15–17). Finally, a convenience sample of 30 cows per herd was constituted for evaluation of hygiene and hock lesions. To be eligible, a cow needed to be lactating and housed on the bedding of interest. In the majority of the recruited herds, the 30 cows sample represented >30% of available cows. In free stall barns, most cows were generally standing when the observer was present in the pen and thus the first thirty cows observed were scored for hygiene and hock lesions. In tie-stall barns, cows from the first thirty stalls were scored. A single trained observer (an animal health technician) did all the evaluations for all farms. The hygiene level of these cows was scored using the Canadian Bovine Mastitis Research Network hygiene chart (2022) (18). This latter chart was adapted from a similar chart developed by the University of Wisconsin-Madison and Pfizer Animal Health. With this chart, cows were assigned a score from 1 to 4 (1 being the cleanest and 4 the dirtiest) for three body regions (udder, lower legs, and flank/upper legs). On the same cows, hocks were scored for presence of lesions. Hocks could be scored from 0 to 3 (Table 1), 0 representing a healthy joint (19). If a hock was so dirty that it was impossible to attribute a score, it was excluded and identified as missing data. For a given cow, the hock with the highest score was used in the model.

**Table 1:** Hock lesions score chart.

**Score^a^**	**Swelling**	**Cutaneous wound**	**Hair loss**
0	None	None	None
1	No swelling or swelling protrusion < 1 cm	None	Bald area
2	Swelling protrusion 1–2.5 cm	May be present	May be present
3	Swelling protrusion >2.5 cm	May be present	May be present

a Reproduced from DFC (19).

### Outcomes studied

We computed hygiene score individually for each body region scored (i.e., one score for udder, one for lower legs, and one for flank/upper legs). For each body zone, we estimated the effect of the bedding type (RMS vs. straw) on three hygiene odds using continuation-ratio models (see details below): (1) odds of having a score ≥2 (compared to a baseline score of 1); (2) odds of having a score ≥3 (compared to scores of 1 or 2) and (3) odds of having a score of 4 (compared to scores of 1, 2, or 3). The effect of the bedding type on the hock score was similarly estimated using a continuation ratio model with three odds: (1) odds of having a score ≥1 (compared to a baseline score of 0), (2) odds of having a score ≥2 (compared to scores of 0 or 1) and (3) odds of having a score of 3 (compared to scores of 0, 1, or 2).

### Statistical analysis

We first generated descriptive statistics for all dependent and independent variables. Then, bivariate relationship between all variables (dependent and independent) were evaluated to detect potential collinearity issues. To describe the association between bedding and the three hygiene scores, we used continuation-ratio models. Continuation-ratio models can be used to model the odds that an individual would move beyond a stage, once a particular stage has been reached. They are, thus, particularly well suited for ordinal categorical outcomes, such as the various health-related scores used in dairy science (20). In our case, the model was designed to evaluate the effect of the bedding type (RMS *vs*. straw) on the odds of being in a specified hygiene level, compared to the odds of being in any of the lower hygiene levels. Two putative confounders previously identified with a directed acyclic graph were included in the models as covariates: housing type (free stall vs. tie-stall), and time since the last renovation of the stalls (in years). Bedding thickness (<10 vs. ≥10 cm of depth) was also identified as a confounder, but could not be included as an independent predictor in the model due to its perfect correlation with housing type and bedding type. Nevertheless, controlling for the first two confounders provided control for the third one, given the perfect correlation between the three confounders. A herd random intercept was included to take into consideration clustering of cows by herd. The continuation-ratio model required three distinct generalized linear mixed models with a binomial distribution and a logit function (20). Using this kind of model, one could estimate the RMS vs. straw-bedding odds ratio of having a specific hygiene score compared with lower hygiene score by exponentiating the bedding variable coefficient.

To estimate the association between bedding type and hock lesion score, a similar continuation-ratio model was used. The only difference was the outcome, which, in this case, was the odds of having a specific hock score compared with lower hock lesion score.

For each generalized linear mixed model used to construct the continuation-ratio models, the linearity assumption of the quantitative predictor (time since the last renovations of the stall) and log odds of the outcomes (hygiene or hock score) was verified by adding polynomial (square or cubic) terms after centering the predictor. Polynomial terms were retained whenever they were significant. Significance level was set at *p* < 0.05. Statistical analyses were carried out with SAS 9.4 (SAS Institute Inc., Cary, NC). Datasets and SAS scripts are publicly available on https://doi.org/10.5683/SP3/ZKBAM4.

## Results

### Description of herds

Herd recruitment was previously described (6, 8, 15–17). Briefly, 49 RMS herds and 139 straw-bedded were contacted by phone to verify if they met the inclusion criteria and their willingness to participate to the study. In the RMS group 11 herds were excluded due to their geographic location, 6 could not be contacted after multiple attempts, 4 had switched to another bedding type and, finally, one did not use the recycled manure solids as bedding for adult dairy cows. All other herds were willing to participate and these 27 herds were, therefore, recruited. In the straw group, the 139 herds contacted had already indicated to their DHI representative that they were willing to participate. Among them, 61 herds were selected on their abilities to provide computerized health data.

**Figure 1:**
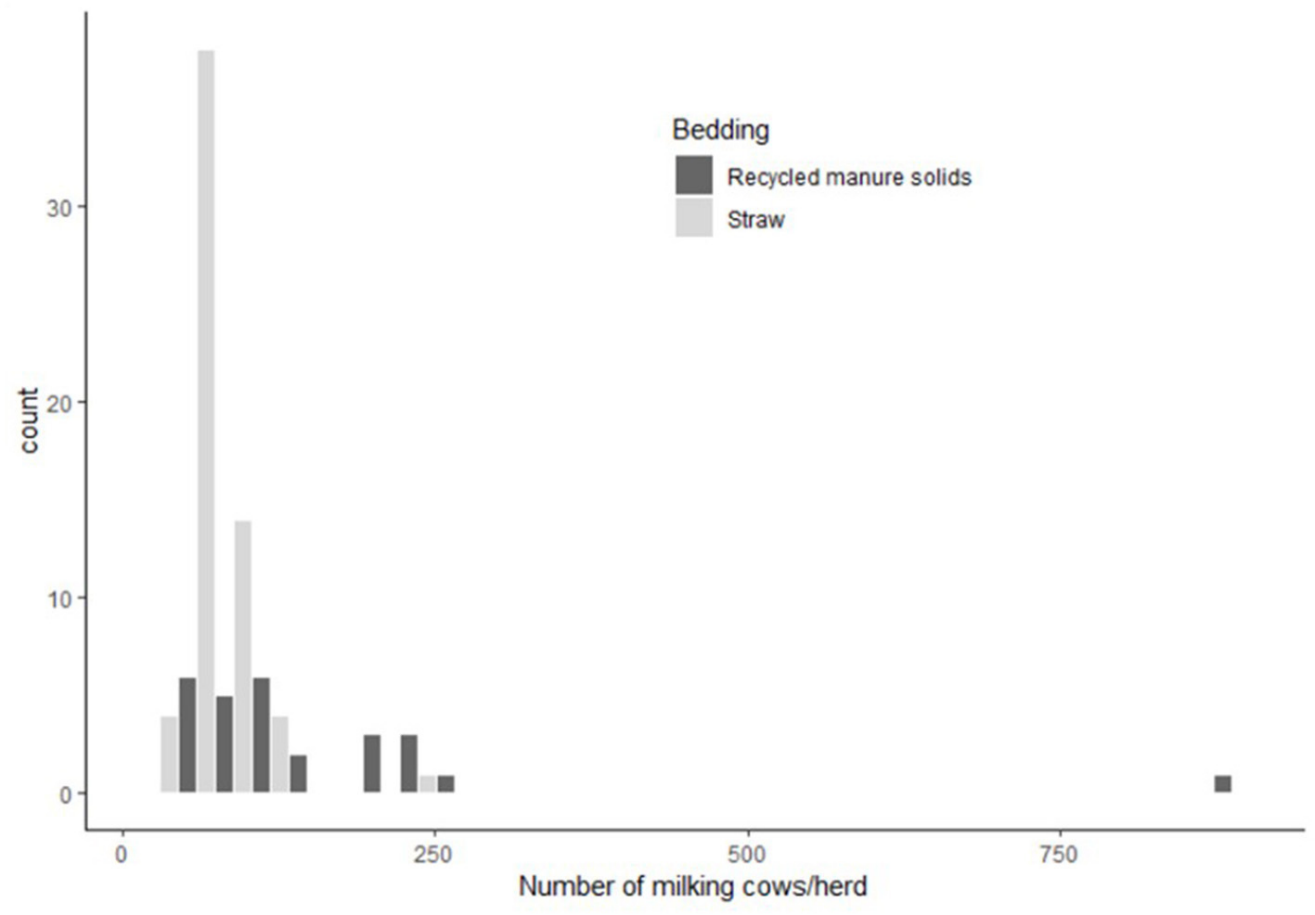
Distribution of number of milking cows in the sampled herds as function of bedding type used (in the histogram, bins with equivalent number of milking cows in recycled manure solids vs. straw-bedded herds are presented next to each other to facilitate viewing).

All farms were visited between January 15th and July 10th 2018. In the RMS group, one herd used an anaerobic digestion followed by a separation to process the solids. One herd used solids right after the separation process (green bedding), 25 others herds used a maturation process after the separation (rotating drum; *n* = 2, maturation in heap; *n* = 10; maturation in closed container; *n* = 13). Length of maturation was highly variable between herds and varied from 0.4 to 9 days (median: 2.5). The median (range) number of months between visit and installation of the RMS system was 18 months (7–112 months). RMS herds were larger than straw herds with a median of 113 vs. 65 milking cows, respectively (Figure 1). The RMS herd size distribution, however, was very skewed due to one very large (*n* = 900 milking cows) herd. Furthermore, RMS farm buildings were more recent, which is demonstrated by the proportion of free stall buildings (RMS: 74% vs. straw: 3%), the number of years since last renovation of the stalls (RMS: 3 vs. straw: 10), and use of deep bedding (RMS: 40% vs. straw: 0%). In the straw group, 36 farms used mattresses as the stall base, 15 used rubber mats, 6 used a combination of mattresses and rubber mats, and 4 farms used a concrete base. In the RMS group, 11 farms used mattresses as the stall base, 11 used a deep bedding system, 3 used a combination of mattresses and rubber mats, and 2 used rubber mats. Straw producers removed manure from stalls more frequently than RMS producers (median 5x/d for straw; range: 1–12 vs. 3x/d for RMS; range: 1–12). Furthermore, bedding was added more frequently to the stalls in straw farms with a median of 2 additions/d (range: 1–4), than in RMS herds in which the median frequency was 1/d (range: 0.14–4).

### Data collection

In total, 2,675 cows were evaluated (843 cows for RMS vs. 1,832 cows for straw). As planned, an average of 30 cows per herd were scored for hygiene and hock lesions (range: 29–33). A small proportion of hocks (3%; 158/5,350) could not be scored for lesions due to their level of soiling. The number of hocks left unscored was not associated with the bedding type (chi-square test; *p* = 0.24). Given the relatively small herd size in the studied population, in >75% of herds, sampling of 30 cows/herd resulted in a sample representing >30% of the available milking cows.

### Hygiene

#### Udder

Udder hygiene scores were similar between the two groups (Table 2), but straw-bedded cows tended to have a numerically larger proportion of score 3 and 4 than RMS herds. The continuation-ratio model could not highlight any statistically significant associations between bedding type and odds of having a score ≥2 (OR RMS vs. straw: 0.62; 95% CI: 0.37, 1.03). However, RMS bedding had a protective effect on the odds of having a score ≥3 (OR: 0.43; 95% CI: 0.20, 0.95) and of having a score of 4 (OR: 0.29; 95% CI: 0.09, 0.93; Table 3).

**Table 2:** Percentage (number) of cow's hygiene score by body region and bedding type obtained on 27 recycled manure solids bedded farms and 61 straw-bedded farms.

**Score^a^**	**RMS bedding**			**Straw bedding**		
	**Body area**			**Body area**		
	**Udder**	**Lower legs**	**Flank/Upper legs**	**Udder**	**Lower legs**	**Flank/upper legs**
1	45.4 (383)	18.4 (155)	33.9 (285)	36.5 (668)	36.4 (666)	27.5 (503)
2	46.1 (389)	67.9 (572)	54.1 (455)	43.0 (787)	50.6 (926)	50.3 (921)
3	7.1 (60)	12.7 (107)	9.3 (78)	13.7 (250)	9.6 (175)	14.1 (259)
4	1.3 (11)	1.1 (9)	2.7 (23)	6.9 (127)	3.6 (65)	8.1 (149)

a Mastitis Network (21).

**Table 3:** Udder hygiene odds ratio comparing cows housed on recycled manure solids bedding to straw-bedded cows, and estimated using continuation-ratio mixed model.

		**Coefficient**	**SE**	***p***	**OR**	**CI^§^**
Odds of score ≥2	Intercept^†^	0.68	0.11			
	Bedding type					
	RMS	−0.48	0.26	0.06	0.62	0.37, 1.03
	Straw	Ref			Ref	
	Housing type^ 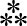 ^					
	Free stall	0.01	0.28	0.96		
	Tie stall	Ref				
	Stall age^ 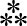 ^	−0.01	0.01	0.15		
	*Variance*					
	Farm	0.38				
Odds of score ≥3	Intercept^†^	−1.28	0.17			
	Bedding type					
	RMS	−0.84	0.4	0.04	0.43	0.20, 0.95
	Straw	Ref			Ref	
	Housing type^ 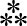 ^					
	Free stall	−0.68	0.44	0.12		
	Tie stall	Ref				
	Stall age^ 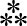 ^	−0.04	0.01	< 0.01		
	*Variance*					
	Farm	0.98				
Odds of score = 4	Intercept^†^	−2.71	0.23			
	Bedding type					
	RMS	−1.25	0.60	0.04	0.29	0.09, 0.93
	Straw	Ref			Ref	
	Housing type^ 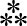 ^					
	Free stall	−0.68	0.66	0.31		
	Tie stall	Ref				
	Stall age^ 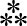 ^	−0.05	0.02	0.02		
	*Variance*					
	Farm	1.31				

§ Confidence interval of the odds ratio (OR).

† Stall age was centered on 5 years.

^
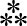
^Putative confounders.

#### Lower legs

For the lower legs hygiene score, RMS-bedded cows had a greater proportion of score 2 than straw-bedded ones (Table 2). The model estimated that RMS had a statistically significant protective effect on the odds of: (1) having a score ≥2 (OR RMS vs. straw: 0.45; 95% CI: 0.21, 0.98); (2) having a score ≥3 (OR: 0.16; 95% CI: 0.04, 0.66); and (3) having a score of 4 (OR: 0.07; 95% CI: 0.01, 0.40; Table 4).

**Table 4:** Lower legs hygiene odds ratio comparing cows housed on recycled manure solids bedding to straw-bedded cows, and estimated using continuation-ratio mixed model.

		**Coefficient**	**SE**	***p***	**OR**	**CI^§^**
Odds of score ≥2	Intercept^†^	0.80	0.17			
	Bedding type					
	RMS	−0.80	0.46	0.04	0.45	0.21, 0.98
	Straw	Ref			Ref	
	Housing type^ 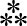 ^					
	Free stall	3.07	0.46	< 0.01		
	Tie stall	Ref				
	Stall age^ 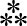 ^	−0.05	0.02	0.02		
	Stall age^2^	7.36E-4	4.32E-4	0.09		
	*Variance*					
	Farm	0.91				
Odds of score ≥3	Intercept^†^	−2.25	0.30			
	Bedding type					
	RMS	−1.82	0.71	0.01	0.16	0.04, 0.66
	Straw	Ref			Ref	
	Housing type^ 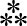 ^					
	Free stall	1.61	0.76	0.03		
	Tie stall	Ref				
	Stall age^ 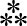 ^	−0.11	0.06	0.07		
	Stall age^2^	3.47E-3	4.43E-3	0.43		
	Stall age^3^	−3.00E-5	5.60E-5	0.59		
	*Variance*					
	Farm	2.66				
Odds of score = 4	Intercept^†^	−3.47	0.28			
	Bedding type					
	RMS	−2.73	0.93	< 0.01	0.07	0.01, 0.40
	Straw	Ref			Ref	
	Housing type^ 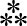 ^					
	Free stall	1.44	0.9	0.11		
	Tie stall	Ref				
	Stall age^ 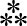 ^	−0.13	0.04	< 0.01		
	Stall age^2^	2.01E-3	7.80E-4	< 0.01		
	*Variance*					
	Farm	1.54				

§ Confidence interval of the odds ratio (OR).

† Stall age was centered on 5 years.

^
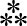
^Putative confounders.

#### Flank/upper legs

Hygiene scores for flank and upper legs were similar between the two farm groups (Table 2). The estimates produced by the continuation-ratio model were all in the same direction, but not statistically significant, with odds of: (1) having a score ≥2 (OR RMS vs. straw: 0.57; 95% CI: 0.30, 1.09); (2) having a score ≥3 (OR: 0.50; 95% CI: 0.20, 1.26); and (3) having a score of 4 (OR: 0.35; 95% CI: 0.11, 1.15; Table 5).

**Table 5:** Flank/upper legs hygiene odds ratio comparing cows housed on recycled manure solids bedding to straw-bedded cows, and estimated using continuation-ratio mixed model.

		**Coefficient**	**SE**	***p***	**OR**	**CI^§^**
Odd of score ≥2	Intercept^†^	1.18	0.14			
	Bedding type					
	RMS	−0.55	0.33	0.09	0.57	0.30, 1.09
	Straw	Ref			Ref	
	Housing type^ 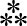 ^					
	Free stall	0.17	0.35	0.63		
	Tie stall	Ref				
	Stall age^ 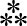 ^	−0.01	9.71E-3	0.18		
	*Variance*					
	Farm	0.64				
Odd of score ≥3	Intercept^†^	−1.36	0.22			
	Bedding type					
	RMS	−0.70	0.47	0.14	0.50	0.20, 1.26
	Straw	Ref			Ref	
	Housing type^ 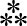 ^					
	Free stall	−0.39	0.51	0.45		
	Tie stall	Ref				
	Stall age^ 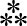 ^	−0.02	0.01	0.11		
	*Variance*					
	Farm	1.49				
Odd of score = 4	Intercept^†^	−2.63	0.25			
	Bedding type					
	RMS	−1.04	0.60	0.08	0.35	0.11, 1.15
	Straw	Ref			Ref	
	Housing type^ 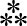 ^					
	Free stall	−0.32	0.65	0.62		
	Tie stall	Ref				
	Stall age^ 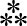 ^	−0.04	0.02	0.05		
	*Variance*					
	Farm	1.63				

§ Confidence interval of the odds ratio (OR).

† Stall age was centered on 5 years.

^
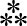
^Putative confounders.

#### Hock lesions

Straw-bedded cows had lower proportion of healthy hocks (score 0) than RMS-bedded cows and had higher proportion of score 2 hocks (Table 6). The continuation-ratio model, however, did not confirm any statistically significant association between bedding type and hock lesions. The odds ratio comparing RMS vs. straw for hock lesion score ≥1 were 0.91 (95% CI: 0.40, 2.07), the odds ratio for score ≥2 where 0.96 (95% CI: 0.53, 1.72) and, finally, the odds ratio for score of 3 were 0.43 (95% CI: 0.11, 1.73; Table 7).

**Table 6:** Percentage (number) of cow's hock lesion scores by bedding type on 27 recycled manure solids bedded farms and 61 straw-bedded farms.

**Score^a^**	**RMS bedding**		**Straw bedding**	
	**Hock**		**Hock**	
	**Left**	**Right**	**Left**	**Right**
0	22.7 (372)	26.2 (432)	11.2 (398)	10.5 (374)
1	42.8 (700)	43.8 (722)	46.0 (1,630)	45.8 (1,628)
2	33.7 (552)	29.1 (480)	42.1 (1,494)	42.3 (1,504)
3	0.7 (12)	1.0 (16)	0.7 (24)	1.3 (46)

a DFC (19).

**Table 7:** Hock lesions odds ratio comparing cows housed on recycled manure solids bedding to straw-bedded cows, and estimated using continuation-ratio mixed model.

		**Coefficient**	**SE**	***p***	**OR**	**CI^§^**
Odds of score ≥1	Intercept^†^	2.81	0.19			
	Bedding type					
	RMS	−0.09	0.42	0.82	0.91	0.40, 2.07
	Straw	Ref			Ref	
	Housing type^ 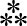 ^					
	Free stall	−0.89	0.44	0.04		
	Tie stall	Ref				
	Stall age^ 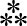 ^	0.06	0.02	< 0.01		
	*Variance*					
	Farm	0.76				
Odds of score ≥2	Intercept^†^	0.13	0.14			
	Bedding type					
	RMS	−0.04	0.30	0.89	0.96	0.53, 1.72
	Straw	Ref			Ref	
	Housing type^ 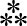 ^					
	Free stall	−0.45	0.33	0.17		
	Tie stall	Ref				
	Stall age^ 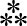 ^	0.03	0.02	0.10		
	Stall age^2^	−2.30E-4	3.51E-4	0.51		
	*Variance*					
	Farm	0.57				
Odds of score = 3	Intercept^†^	−4.22	0.28			
	Bedding type					
	RMS	−0.85	0.71	0.23	0.43	0.11, 1.73
	Straw	Ref			Ref	
	Housing type^ 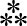 ^					
	Free stall	0.66	0.73	0.36		
	Tie stall	Ref				
	Stall age^ 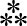 ^	−3.70E-3	0.02	0.85		
	*Variance*					
	Farm	0.99				

§ Confidence interval of the odds ratio (OR).

† Stall age was centered on 5 years.

^
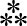
^Putative confounders.

## Discussion

When adjusting our estimates for type of farm (tie-stall vs. free stall) and time since the last renovation of the stalls, we observed that using RMS bedding had, generally, a protective effect for udder and lower legs hygiene and no effect on flank/upper legs hygiene. This result differs from previous studies who have not observed differences (10–12) in cows' hygiene between beddings. This difference in results may be partly explained by the inclusion of putative confounders (housing type and stall age) in our statistical models, while previous studies did not report accounting for confounding in their study design nor analyses. For instance, in our study, not adjusting the bedding and udder hygiene relationship for confounding was biasing our OR estimates by −23 to +9% (data not shown). Similarly, for the bedding and lower leg hygiene relationship, not adjusting for confounding was creating biases of the OR of +80% (data not shown). The important magnitude of this latter bias is expected, since housing system (tie-stall vs. free stall) is strongly associated with lower leg hygiene and, in our study, with bedding type. A last design characteristic that could explain the differences between our study's conclusions and those from previously published literature, is the use of continuation-ratio models in our study, thus accounting for the four hygiene score categories, while, in previous literature, this score was most often dichotomized in two categories. Nevertheless, none of the previously mentioned studies identified RMS as being worse for hygiene level in cows, which agrees with our findings.

It is interesting to note that, even if the RMS-bedded cows studied had visually cleaner udders, they did not have lower clinical mastitis incidence (6). Conversely, the cows studied in the current research project were at much greater risk (7 times) of having clinical mastitis due to *Klebsiella pneumoniae*, than cows housed on straw bedding (6). Moreover, in these same herds, we found similar and sometimes lower counts of bacteria (including *Klebiella* spp) in unused and used RMS vs. straw bedding (16). Thus, the higher *Klebsiella pneumoniae* clinical mastitis incidence observed in RMS herds does not seem to be associated with an increased amount of this bacterial species in the bedding, nor with an increased dirtiness of the udder. A potential hypothesis is that this bedding type would stick more readily to the teat end, and, thus, would promote a more efficient and longer exposure of the teat end to *Klebsiella* spp. In the current study, it would have been interesting to collect teat swabs to investigate the relationship between bacterial populations in bedding, visual udder hygiene, and teat skin pathogen populations before milk harvesting. This would have improved our understanding of this important health issue.

The lower legs cleanliness of RMS-bedded cows was unexpected. In another research, it was also found that cows housed on RMS in an experimental tie-stall farm had a wetter and softer sole than cows housed on straw (Villettaz-Robichaud, personal communication). For cows housed in tie-stall farms, where they spend all their time on this surface, this could potentially lead to sole diseases and this should be investigated in future research.

The prevalence of hock lesions was not different between the two groups. In the literature, RMS-bedded cows had, generally, poorer hock lesions scores than cows housed on sand bedding. However, if we look at performance of straw bedding specifically, some research estimated that its usage was leading to more hock lesions than RMS (13), while others concluded the opposite, with RMS-bedded cows being more at risk of severe hock lesion (11). Differences between studies may be partly due to inclusion, or not, of putative confounders related to housing type, floor surface, or stall design.

Management of hock lesions is challenging and it was demonstrated that usage of deep-bedding systems results in a decrease of hock lesions prevalence (14, 22). Others also demonstrated that bedding depth, by mitigating the impact of abrasive or hard stall base on cows, may play a more important role for the cow welfare than the bedding type itself (2). Due to its large availability once a farm is equipped, RMS is of great interest since it could be used in large amount in stalls, including deep-bedded stalls. However, RMS is considerably less dense than sand, another common bedding type used in deep bedding system (14). Therefore, when it will be used, RMS will tend to be compressed more easily than sand, which may expose the rear curb of the stalls and cause an increase in hock lesions prevalence. The latter was indeed reported with sawdust bedding in deep-bedding systems (23). This potential negative impact could be attenuated with frequent addition of bedding in stalls (21, 24). When compared to other bedding types, RMS may also be problematic for hock's health in regards to its low dry matter content. Prolonged contact with wet bedding may compromise the integrity of the skin, which may then be prone to the development of lesions (23, 25). Finally, in our study, we found that cows housed on RMS had cleaner lower legs than cows housed on straw. While having animals with a good hygiene level is desirable, this may put them at a greater risk of developing hock lesions. As a matter of fact, dirt could serve as a sort of protective shield for the skin (25, 26).

The decision of evaluating the cow comfort based on the presence of hock lesions was in a large part driven by the study design (a large number of herds and cows to follow vs. what could have been achieved in an experimental design). Presence of hock lesions is not a robust measure of comfort, and it may be affected by several other aspects of the stalls' design (2, 25). Time since the last renovations of the stall was controlled in the model to reduce the possible impact of old, poorly designed stalls. Other factors could have been evaluated to measure the comfort, such as the number of lying bouts or duration of lying bouts (2, 27). However, recording these measures is not easily achievable in observational studies conducted on a large number of commercial farms. In an experimental study carried out in Québec (2018), the total lying time per 24 h was not different between cows housed on straw vs. RMS (Villettaz-Robichaud, personal communication).

### Study strengths and limitations

Another important confounder of the association between bedding type and hock lesions is bedding thickness (< 10 vs. ≥ 10 cm of depth). However, due to severe collinearity issues, bedding thickness could not be included in the model. Nevertheless, it was totally corrected by the inclusion of farm housing type, since deep-bedding systems were only found in free stall farms.

Use of a continuation-ratio model is a strength of this study and has allowed us to study each score variation (odds of being in a category to the probability of being in any lower category) and its relation with bedding type (20). With ordinal data (e.g., hygiene or hock scores) the use of linear regression models would not be appropriate, since scores are not quantitative variables (i.e., an increase of one unit, does not necessarily represent the same fixed quantity when going from a score of 1 to a score of 2 vs. when going from a score of 2 to a score of 3). The other approach that was often used in the literature was to dichotomise the multinomial score into two categories (e.g., 1–2 vs. 3–4). But this latter approach tends to oversimplify the outcome. Indeed, why would we use four points scoring system if the issue at end was truly a yes/no answer? There is a gradation when measuring hygiene, hock lesions, and many other animal scores. The continuation-ratio is quite a simple and efficient method for maintaining this gradation in the analysis phase.

On the other hand, our project presents some limitations due to the observational design. Herds using RMS or straw bedding were selected using different methods; mainly a census of available herds for RMS vs. a selection within DHI participating herds for straw. Nevertheless, 80% of RMS herds were also participating in regular DHI monitoring, thus limiting a potential selection bias. Besides, residual confounding may still be present, although we adjusted our models for the confounders we think had the most impact on the measured associations. Other unmeasured confounders may distort associations in an unpredictable direction and amplitude. Furthermore, the generalizability of our results may be limited to the Quebec province due to the sample population characteristics (predominance of tie-stall farms, small/medium sized herds). Besides, an experimental design would be of great interest to confirm our results, but is unlikely to be conducted given the cost of equipment and the installations required. Another option would be to follow farms transitioning to RMS bedding and record data on health before and after the transition. With time, more studies will be available and meta-analysis or literature reviews of these studies will help us contextualize our results on a larger scale.

Finally, it is important to emphasize that many other, and, perhaps, more important factors can affect hygiene of dairy cows and/or hock lesions prevalence (25). For instance, width of the passageways, stocking density, cleaning frequency could all have greater impact on cows' hygiene than the actual type of bedding used. Similarly, stall design (length, width, position of brisket board or neck rail, lunge space, curb, etc.) certainly has a greater impact on risk of developing hock lesions than bedding type (2).

## Conclusion

In herds in Quebec, cows housed on RMS bedding had, generally better udder and lower legs hygiene than straw-bedded ones. No difference was found between the two bedding types regarding hygiene of the flank/upper legs body region. Furthermore, no difference in odds of hock lesions was detected between RMS-bedded cows and straw-bedded ones.

## Data availability statement

The datasets presented in this study can be found in online repositories. The names of the repository/repositories and accession number(s) can be found below: https://doi.org/10.5683/SP3/ZKBAM4.

## Ethics statement

The animal study was reviewed and approved by Animal Care and Use Committee of the Faculté de Médecine Vétérinaire (Université de Montréal; protocol 17-Rech-1886). Written informed consent was obtained from the owners for the participation of their animals in this study.

## Author contributions

SD and GF designed the study. AF collected and analyzed data with SD supervision. AF wrote the first draft of the paper which has been subsequently reviewed and approved by GF and SD. All authors approved the final version of the paper before the submission process.
